# *Bacillus anthracis* Phylogeography: New Clues From Kazakhstan, Central Asia

**DOI:** 10.3389/fmicb.2021.778225

**Published:** 2021-12-08

**Authors:** Alexandr Shevtsov, Larissa Lukhnova, Uinkul Izbanova, Jean-Philippe Vernadet, Marat Kuibagarov, Asylulan Amirgazin, Yerlan Ramankulov, Gilles Vergnaud

**Affiliations:** ^1^National Center for Biotechnology, Nur Sultan, Kazakhstan; ^2^National Scientific Center for Especially Dangerous Infections Named by Masgut Aykimbayev, Almaty, Kazakhstan; ^3^CEA, CNRS, Institute for Integrative Biology of the Cell (I2BC), Université Paris-Saclay, Gif-sur-Yvette, France; ^4^School of Science and Humanities, Nazarbayev University, Nur Sultan, Kazakhstan

**Keywords:** *Bacillus anthracis*, whole genome sequencing (WGS), MLVA (multiple locus variable number tandem repeats analysis), phylogeography, single nucleotide polymorphism (SNP), evolution, Paleolithic, Neolithic

## Abstract

This article describes *Bacillus anthracis* strains isolated in Kazakhstan since the 1950s until year 2016 from sixty-one independent events associated with anthrax in humans and animals. One hundred and fifty-four strains were first genotyped by Multiple Locus VNTR (variable number of tandem repeats) Analysis (MLVA) using 31 VNTR loci. Thirty-five MLVA31 genotypes were resolved, 28 belong to the A1/TEA group, five to A3/Sterne-Ames group, one to A4/Vollum and one to the B clade. This is the first report of the presence of the B-clade in Kazakhstan. The MLVA31 results and epidemiological data were combined to select a subset of seventy-nine representative strains for draft whole genome sequencing (WGS). Strains from Kazakhstan significantly enrich the known phylogeny of the Ames group polytomy, including the description of a new branch closest to the Texas, United States A.Br.Ames sublineage stricto sensu. Three among the seven currently defined branches in the TEA polytomy are present in Kazakhstan, “Tsiankovskii”, “Heroin”, and “Sanitary Technical Institute (STI)”. In particular, strains from the STI lineage are largely predominant in Kazakhstan and introduce numerous deep branching STI sublineages, demonstrating a high geographic correspondence between “STI” and Kazakhstan, Central Asia. This observation is a strong indication that the TEA polytomy emerged after the last political unification of Asian steppes in the fourteenth century of the Common Era. The phylogenetic analysis of the Kazakhstan data and of currently available WGS data of worldwide origin strengthens our understanding of *B. anthracis* geographic expansions in the past seven centuries.

## Introduction

*Bacillus anthracis*, a soil-borne bacterial species causing anthrax in animals and humans, combines two virulence plasmids, pXO1 and pXO2 and a *Bacillus cereus sensu lato* chromosomal background (Okinaka and Keim, 2016; Pena-Gonzalez et al., 2018). Anthrax mentioned in early historical records was among the diseases of high consequences for livestock and humans until the end of the nineteenth century when its causes and the ecology of the pathogen were sufficiently understood. Subsequently prophylactics (efficient vaccines and carcasses disposal procedures) and therapeutics (antibiotics) were developed (Klemm and Klemm, 1959; Hugh-Jones and De Vos, 2002; World Health Organization [WHO], 2008) so that the disease is now globally controlled. The potential use of *B. anthracis* as a bioweapon has, however, been the source of new concerns and has encouraged the development of forensic tools (Keim et al., 2004; Takahashi et al., 2004; Sahl et al., 2016). It is hoped that “microbial forensics” capacities to trace back the source of an attack, might act as a deterrent vis-à-vis potential users (Schmedes et al., 2016). This goal has had a major impact regarding our knowledge of the genetic diversity of *B. anthracis*, and our capacity to investigate its phylogeography.

The classical view regarding the origin and spread of *B. anthracis*, as reflected for instance by Kolonin (1971), is that the bacteria was already present across Eurasia and Africa during the Paleolithic, tens of thousands of years before the start of the Neolithic period. Climate changes, landscape including soil composition and human activities (domestication of animals, migrations) would have modulated the current distribution of a pre-existing pathogen, explaining the relative prevalence of distinct lineages (Van Ness, 1971; Smith et al., 2000).

In the past twenty years, the advent of genetic tools including the emergence of highly efficient genotyping and whole genome sequencing led to the description of the population structure of *B. anthracis*. Firstly, the analysis of polymorphic tandemly repeated DNA sequences allowed to define three lineages, called A, B, and C (Keim et al., 2000). Branch A is predominant worldwide whereas B is much more restricted. Branch B has been found mostly in Northern Eurasia, in mountainous areas across Western Europe, in South Africa and United States (Keim et al., 2000; Fouet et al., 2002; Timofeev et al., 2019; Eremenko et al., 2021). Branch C is extremely rare with only a few known representatives, isolated in the United States. The evolution of the *B. anthracis* genome was subsequently shown to be strictly clonal, and the species is highly homogeneous (Pearson et al., 2004). Keim and colleagues took advantage of available whole genome sequence data and whole genome Single Nucleotide Polymorphism (wgSNP) analysis to propose a first dating of the divergence of known lineages. The calculation resulted in an estimate of 25,000-50,000 years before present (ybp) for the age of the most recent common ancestor (MRCA) of *B. anthracis* lineages and of 6,000 to 13,000 ybp for the primary radiation within the A-lineage (Van Ert et al., 2007; Kenefic et al., 2009). This estimate has many uncertainties discussed in detail by the authors, in particular the estimation of an average number of infections per year but it was compatible with the classical view of a Paleolithic pathogen. *B. anthracis* might be much older than the MRCA, and the primary radiation within the A-lineage would reflect the effect of anthropic activities on the pre-existing Paleolithic canvas. The estimation of the origins for North American Anthrax comforted this view by the suggestion that the main ecologically established lineage in North America, called WNA (western North America) arrived more than 13,000 ybp *via* the Bering Land Bridge (Kenefic et al., 2009).

This analysis was recently challenged owing to the production in the past ten years of extensive WGS data worldwide. The new data allowed to pinpoint the emergence of WNA in the phylogenetic tree of *B. anthracis*. The WNA lineage emerged from a branch so far found exclusively in France, Spain and Italy (Vergnaud et al., 2016; Vergnaud, 2020), providing strong evidence in favor of a post-Columbian contamination of North America a few centuries ago instead of more than 10,000 ybp. This revised dating opens the possibility of a Neolithic origin for *B. anthracis* (Vergnaud, 2020). Under the proposed Neolithic scenario, the phylogenetic tree is structured along a backbone going from an hypothetical cradle in Central Africa suggested by the discovery of *B. cereus* biovar *anthracis* carrying both virulence plasmids (Hoffmaster et al., 2004; Leendertz et al., 2004; Klee et al., 2006; Antonation et al., 2016; Pena-Gonzalez et al., 2018; Baldwin, 2020) to the unique *B. anthracis* lineage present almost exclusively in Africa, “AncientA” (Sahl et al., 2016). Under this model, lineages A, B, and C constitute successful Out-of-Africa exports of *B. anthracis* (Vergnaud, 2020).

Owing to its strict clonality, the population structure of *B. anthracis* is conveniently reflected by canonical Single Nucleotide Polymorphism (canSNP) assignment (Keim et al., 2004; Van Ert et al., 2007; Sahl et al., 2016). Alternative schemes have recently being proposed, including a core genome multilocus sequence typing (cgMLST) approach benefiting from software developed for recombining pathogens (Abdel-Glil et al., 2021) or even a complete renaming inspired by the nomenclature used for *Mycobacterium tuberculosis* (Bruce et al., 2020). While the cgMLST approach may be convenient for the automated, first-level assignment of whole genome sequence data, the proposition of a full renaming does not bring detectable added value compared to the currently used nomenclature (Sahl et al., 2016). Lineage A, the most widespread lineage, comprises the Vollum, Sterne/Ames, Australia94, V770 and the trans-Eurasian (TEA) groups. TEA includes seven monophyletic groups (Tsiankovskii, STI, Pasteur, Heroin, TEA 011, A0150, and A0245). TEA 011 itself is a seven-branches polytomy (Vergnaud, 2020). One branch gave birth to WNA present almost exclusively in North America (Kenefic et al., 2009). Each of these lineages shows some level of geographic association, often blurred by numerous more or less successful export events. For instance, strains from group Australia94 were first identified in Australia but the group is also predominant in East Anatolia and South Caucasus (Khmaladze et al., 2014; Sahin et al., 2018; Eremenko et al., 2021), and Australia is believed to have been contaminated in 1847 *via* bones imported from India and used as fertilizer (Davies and Harvey, 1972; Van Ert et al., 2007; Barro et al., 2016; Muller et al., 2020). The Neolithic model predicts that world history will allow to infer dating points along the phylogeny, in addition to the WNA branching point from Europe toward North America or the “Australia94” export to Australia. However, sequencing data regarding the strains present in many countries is still lacking. Such data is necessary to evaluate the proposed hypotheses.

Anthrax is enzootic in Kazakhstan, Central Asia (Woods et al., 2004; Kracalik et al., 2012). Currently 2433 epizootic foci with records of disease of farm animals, 1516 burial sites, and 1778 settlements classified as permanently unfavorable because of anthrax are registered. All 14 regions of Kazakhstan are concerned. From 1935 to 2018, 1909 human patients were diagnosed with anthrax. Sixty-six cases were fatal. During this time span, 25344 cases of diseases of domestic and commercial animals kept in a household or farm were registered, including 17858 sheep, 4802 cattle, 1514 horses, 1056 pigs, eight camels, two dogs and 104 fur-bearing animals (Arctic Fox, mink). Over the past twenty years, small cattle accounted for 70.5% of the total number of recorded cases of animal disease. Cattle accounted for 19%, horses for 6%, and swine for 4.2%. The total share of other animals did not exceed 0.4%. However, the involvement of different animal species in outbreaks does not correlate with the number of infected animals. Since 1961, cattle have accounted for 45 to 54% of outbreaks, while outbreaks involving small cattle have accounted for 25 to 38%. Isolated cases predominate in outbreaks among cattle and horses, while the average number of diseased animals is higher in outbreaks among sheep and pigs (Kracalik et al., 2012). Three cases of death from anthrax in the wild were officially registered: foxes in the Almaty region in 1980 and two fatal cases of roe deer in the Kyzylorda region in 1951 and 1957. These data do not reflect the true picture of animal morbidity in the wild, since monitoring and bacteriological examination of the remnants are not carried out (Lukhnova et al., 2018; Lukhnova, 2019).

A previous report (Aikembayev et al., 2010) included 89 *B. anthracis* strains collected in Kazakhstan up to year 2005. The strains were characterized by Multiple Locus VNTR (Variable Number of Tandem Repeats) Analysis (MLVA) using eight loci and by canSNP analysis (Keim et al., 2000, 2004). All strains were shown to belong to Branch A, with a predominance of MLVA group A1a corresponding to TEA. A few strains were assigned to MLVA clade A3b corresponding to canSNP Sterne/Ames, and MLVA clade A4 (canSNP Vollum) (Aikembayev et al., 2010; Mullins et al., 2011). In the present report, we expand the previous work by including strains collected up to year 2016 and characterized the 154 strains by an MLVA assay comprising 31 loci (Beyer et al., 2012; Thierry et al., 2014). Importantly, because precise phylogenetic investigation requires the full resolution provided by whole genome sequence (WGS) data, we then sequenced a subset of 79 representative strains. We compared the obtained data with publically available *B. anthracis* WGS data.

## Materials and Methods

### Strains Origin and DNA Extraction

One hundred and fifty-four strains deposited at the National Scientific Center for Especially Dangerous Infections named after Masgut Aykimbayev (NSCEDI) were available for analysis, including one strain collected in 1978 in neighboring Kyrgyzstan (Supplementary Table 1). Isolation of the causative agent of anthrax was carried out in BSL-3 level laboratories in accordance with the current order of the Republic of Kazakhstan dated October 1, 2004 No. 725 and the Minister of Agriculture of the Republic of Kazakhstan dated October 7, 2004 No. 575 “On strengthening measures to prevent anthrax in the Republic of Kazakhstan.” Strains isolated from humans were anonymized, and their use was approved by the local ethics committee of the National Center for Biotechnology (Protocol # 1 from 21.01.2020). The strains were stored in lyophilized or cryopreserved forms.

The collection covered 61 independent events associated with anthrax in humans and/or animals. Eighty-nine strains from 50 outbreaks were collected between 1952 and 2005 and were previously investigated using MLVA8 and canSNPs (Aikembayev et al., 2010). Sixty-five strains from eleven outbreaks were collected between 2006 and 2016. The STI vaccine strain obtained in 1940 at the Sanitary Technical Institute in Tverskaya area was included as control. Ten regions are represented. Turkistan, East Kazakhstan, Jambyl and West Kazakhstan with 26, 9, 7 and 7 events, respectively, contribute more than 80% of the events (Supplementary Table 2), in agreement with the known epidemiology of anthrax in Kazakhstan (Kracalik et al., 2012). In 29 events, the host was large cattle, whereas in two events small cattle were involved. In two events, both categories were affected. In the other 28 events, the information was not available (Supplementary Table 1). Forty-two events were represented by a unique strain whereas 19 events numbered Event01 to Event19 were represented by two up to 12 strains for a global amount of 112 strains (Supplementary Tables 1, 3). Sixty strains were isolated from sick people, 44 from cattle, 16 from sheep, three from horse, one from camel. Twenty-two and three strains were isolated from soil and manure from the place of keeping or forced slaughter of sick animals, respectively; two strains were isolated from cooked cattle meat; two strains were isolated from a shop or sausage factory, and one strain was isolated from a door handle (Supplementary Table 1).

DNA was isolated using the QIAamp DNA Mini Kit (Qiagen, United States) from the inactivated bacterial mass as described earlier (Shevtsov et al., 2019).

### Multiple Locus Variable Number of Tandem Repeats Analysis Typing and Selection of Representative Strains for Whole Genome Single Nucleotide Polymorphism Analysis

*In vitro* MLVA genotyping was performed using 31 VNTR loci (MLVA31) as previously described (Beyer et al., 2012; Thierry et al., 2014; Shevtsov et al., 2019). MLVA genotypes were compared to published data by using the *Bacillus anthracis* MLVA database at https://microbesgenotyping.i2bc.paris-saclay.fr/.

We subsequently selected one strain from each genotype in the sixty-one events (except for KZ148 Event 35, in which case MLVA31 genotype 5 was already represented by seven strains from six different events, Supplementary Figure 1). Seven events (events 01, 03, 07, 10, 11, 15, 17) comprised two MLVA31 genotypes, and two events (events 04 and 12) comprised three MLVA31 genotypes, resulting in the selection of 71 strains (Supplementary Table 1). As controls, we also included eight strains redundant in terms of epidemiology and MLVA31 genotype (Supplementary Figure 1). These controls comprised three strains from Event10, two of which are negative for the pXO1 VNTR locus but are otherwise identical to all the other Event10 strains in terms of MLVA31. In total, 79 strains were selected for draft whole genome sequencing (Supplementary Figure 1 and Supplementary Table 1).

### Genome Sequencing and Single Nucleotide Polymorphism Calling

Sequencing libraries were prepared using Nextera XT DNA Library Preparation Kit (Illumina, CA, United States). Sequencing was performed using MiSeq Reagent Kit v3 (600 cycles) on the Illumina MiSeq platform. Single Nucleotide Polymorphisms (SNPs) were called by mapping raw sequencing reads on Ames ancestor reference genome assembly accession GCA_000008445.1 using BioNumerics version 7.6.3 (Applied-Maths, Laethem-Saint-Martin, Belgium) as previously described (Timofeev et al., 2019). For comparison, publicly available assemblies and raw reads were downloaded *via* EBI-ENA (last update August 31st 2021). Assemblies were split into 50bp long artificial reads, which were then used for SNP calling. Lineage assignments follow the global nomenclature based on selected SNPs (Sahl et al., 2016). BioNumerics was used for Maximum Parsimony analysis and dendrogram drawing. Sequence data of three strains representing Event13 and Event14 were independently reported (Shevtsov et al., 2020). Raw sequencing data were deposited in the EBI archive repository Bioproject PRJNA639508 accessible at https://www.ebi.ac.uk/ena/browser/view/PRJNA639508. Individual sequence reads archives (SRA) accessions are also indicated in Supplementary Table 1.

## Results and Discussion

### MLVA31 Analysis

MLVA31 resolved 35 MLVA31 genotypes consistent with previous MLVA8 results (Keim et al., 2000; Aikembayev et al., 2010) (Supplementary Figure 1 and Supplementary Table 1). The Simpson’s diversity index of MLVA31 is 0.95. Most loci are only moderately polymorphic in this collection, and a more cost-efficient assay appropriate for Kazakhstan could be devised using ten loci as previously proposed (Thierry et al., 2014; Shevtsov et al., 2019). One hundred and thirteen strains can tentatively be assigned to clade A1b_TEA, 27 to A3b_Sterne/Ames and two strains to A4_Vollum. All twelve strains from the 2016 Event19 show the same MLVA31 genotype tentatively assigned to B1_B.Br.001/002. This is to our knowledge the first report of a B-clade strain in Kazakhstan. The outbreak occurred in Uzynsu village, Ertis district, Pavlodar region. There is no registered history of anthrax in this village, located a few kilometers south from the Russian border, suggesting that this lineage is not ecologically established in the area.

### Congruence Between Multiple Locus Variable Number of Tandem Repeats Analysis Clustering and Whole Genome Single Nucleotide Polymorphism Analysis

Supplementary Figure 2 illustrates the congruence between MLVA31 and wgSNP with one minor exception: the three MLVA31 genotypes constituting MLVA31 clonal complex CC01 (Supplementary Figure 1 MLVA31 genotypes 1, 2, and 3) belong to different “STI” sublineages separated by almost 100 SNPs. The eight redundant control strains (black dots) are located at zero up to five SNPs from their “sister” strain (same outbreak and same MLVA31 genotype) as is typical for strains from the same outbreak (Vergnaud, 2020; Abdel-Glil et al., 2021). In the five events (open dots, events 04, 10, 11, 15, 17) showing two MLVA31 genotypes differing at only one locus, the strains are less than three SNPs apart. The five other cases (colored dots, events 01, 03, 04, 07 and 12) with two MLVA31 genotypes differing by two up to five VNTR loci are confirmed as originating from clearly different lineages by wgSNP (a few tens of SNPs differences). The information available for these five outbreaks is compatible with the presence of two different lineages as detailed in Supplementary Table 3.

### Positioning of Strains From Kazakhstan Within the Global *Bacillus anthracis* Population

By combining the MLVA31 data of all 154 strains from 61 events and the wgSNP analysis of the 79 representative strains, 27 strains can be assigned to Sterne/Ames, two strains to Vollum, and twelve strains to the B-clade. The TEA clade represented by 113 strains is largely predominant. Altogether, 54 events are associated with strains from TEA, four events with Sterne/Ames, two events with Vollum and one with B.Br.001/002 (Supplementary Tables 1, 2).

Three among the seven currently defined TEA branches (Sahl et al., 2016; Timofeev et al., 2019) are present in Kazakhstan: 93 strains from 47 events are assigned to lineage “STI” (containing the STI vaccine strain), 14 strains from five events are assigned to lineage “Tsiankovskii” (containing the Tsiankovski 1 vaccine strain), and six strains from five events are assigned to lineage “Heroin.”

#### Kazakhstan Is Predominantly Occupied by the Sanitary Technical Institute Lineage

**FIGURE 1:**
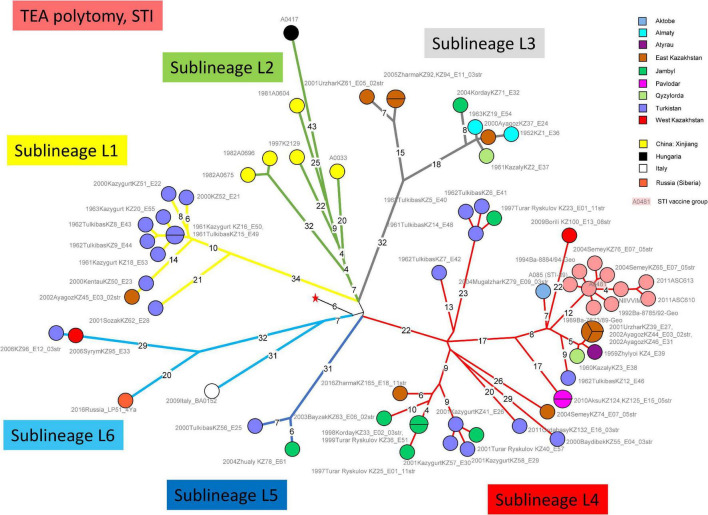
Whole Genome Single Nucleotide Polymorphism (wgSNP) analysis of strains assigned to TEA clade “Sanitary Technical Institute (STI).” Maximum parsimony analysis. 859 SNPs in tree, tree size 867 (homoplasia 0.9%). Branch lengths of four SNPs and more are indicated, a linear scale was used. The red star indicates the position of the root (MRCA) of the TEA polytomy. Each strain is labeled with year of isolation, place (district level or country), strain Id, event number, number of associated strains when above one. Color code reflects the geographic origin of the strains. The STI vaccine strain A0481 is shadowed in pink. Six deep-branching sublineages are defined and branches are colored accordingly.

**FIGURE 2:**
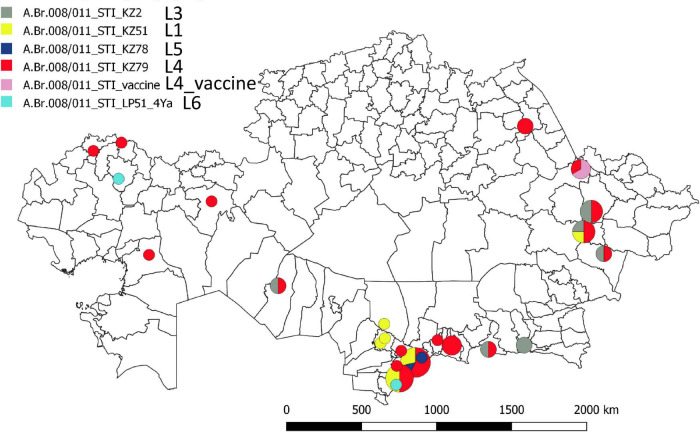
Geographic distribution of the STI sublineages across Kazakhstan. The geographic location of the fifty representative STI strains is indicated at district level resolution. The color code reflects the assigned STI sublineage. Turkistan (South Kazakhstan) region next to the ancient trade routes shows the highest concentration and genetic diversity.

Figure 1 shows a maximum parsimony tree deduced from wgSNP data including strains from Kazakhstan and of worldwide origins assigned to lineage STI. Six deep-branching sublineages can be defined. Sublineage STI_L1 shows a strong geographic association with Turkistan, where it was already present in 1961. The only exception to this geographic association is strain KZ45 recovered in East Kazakhstan from a hide of unknown origin. Sublineage STI_L4 was the most frequently observed. It is characterized by the presence of a polytomy with three branches. Two of these branches show a strong geographic association with Turkistan and the adjacent Jambyl. The third branch of STI_L4 is the most geographically diverse. It includes strains isolated in seven regions of Kazakhstan and the STI vaccine strain (Figure 2). Sublineage STI_L6 is defined by two strains from West Kazakhstan and Turkistan, one strain recovered from permafrost in Northern Siberia (Timofeev et al., 2019) and one strain from Italy. Sublineage STI_L2 is the only one not observed in Kazakhstan. It comprises strains isolated in China, with one exception from Hungaria. This is in agreement with studies describing the genetic diversity of *B. anthracis* in China, and the finding of TEA in the Xinjiang province in Western China (Wang et al., 2020).

This geographic distribution of the STI lineage is quite remarkable. It apparently does not expand eastward beyond the Xinjiang autonomous region in China (Simonson et al., 2009; Wang et al., 2020). Its expansion south of Kazakhstan (Uzbekistan, Turkmenistan, Kyrgyzstan, and Tajikistan) is unknown. Eremenko et al. described 66 strains from European Russia (36 strains) or Asian Russia (Siberia, 30 strains) (Eremenko et al., 2021). Sequence data was not made public and consequently we could not directly include the strains in the present analyses. However, the authors provided a list of SNPs allowing to assign the strains at sublineage level. Six among the thirteen strains assigned to STI are vaccine strains. Four others correspond to the same outbreak event in Dagestan (Caucasus) and belong to sublineage STI_L6. The last three originate from Eastern Siberia and belong to sublineage STI_L2 (strain I-361) or sublineage STI_L4 (strains I-63 and I-356). Consequently, STI sublineages L1, L3, and L5 have not yet been observed outside of Kazakhstan.

#### The “Tsiankovskii” Lineage Presence in West Kazakhstan Is Monophyletic

**FIGURE 3:**
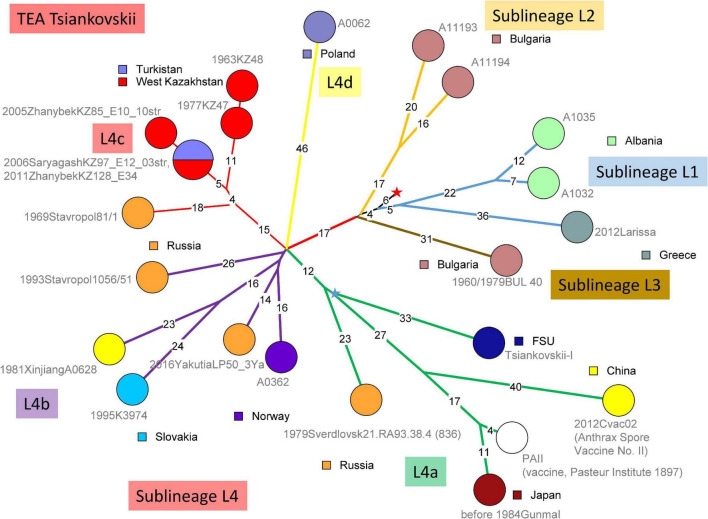
wgSNP analysis of strains assigned to TEA clade “Tsiankovskii.” Maximum parsimony analysis. 587 SNPs in tree, tree size 588 (homoplasia 0.17%). Branch lengths of three SNPs and more are indicated, a linear scale was used. Each strain is labeled with year of isolation, geographic origin, strain Id, event number when context information is available, number of associated strains when above one. Color code reflects the geographic origin of the strains. The red star indicates the position of the root (MRCA) of the TEA polytomy. Four deep-branching sublineages are defined. The four-branches polytomy within sublineage 4 are lettered [“a” to “d”; strain 506-55 (Eremenko et al., 2021) may constitute a fifth branch]. Branches are colored accordingly. Branch L4a contains the Tsiankovskii-I vaccine strain itself. The Tsiankovskii-I vaccine strain was developed in 1883 (Marinin et al., 2008; World Health Organization [WHO], 2008). Tsenkovsky developed the vaccine in Kharkov, in the then-Russian Ukraine (Fedotova, 2012). Branch L4a contains additional vaccine strains, PAII used in Japan (Okutani et al., 2019) and Cvac02 used in China (Zhou et al., 2015). The topology of branch L4a strongly suggests that PAII and Cvac02 are both derived from the Tsiankovskii vaccine. The blue star reflects the position of an hypothetical Tsiankovskii-I ancestor strain (the MRCA of the vaccine associated branches). The weaponized Sverdlovsk strain (Sahl et al., 2016) is branching two SNPs prior to this MRCA. Branch L4b represented by five independent strains, is predominantly European, with one exception from Western China. It contains two strains recovered from high latitudes, one in Norway and the other from Yakutia, Siberia (Timofeev et al., 2019). Nine strains from Eremenko et al. (2021) can be assigned to this branch. Branch L4c is present in the Stavropol area and in West Kazakhstan. Ten strains from Eremenko et al. (2021), including published Stavropol strain 81/1 belong to Tsiankovskii branch L4c. These strains constitute a polytomy with four branches (Eremenko et al., 2021). The strains from Kazakhstan branch out from one of these four branches. The most parsimonious interpretation of the observed topology is the introduction of a single lineage from Southern Russia to West Kazakhstan. Finally, lineage L4d is represented by a single strain from Poland. Two strains from Republic of Moldova (strains 802-263 and 575-122) might also belong to Tsiankovskii L4d according to the published list of SNPs (Eremenko et al., 2021).

Figure 3 shows the topology of the Tsiankovskii branch within the TEA polytomy. Starting from the root indicated by the red star, four sublineages split within five SNPs distance. Sublineage L1 was observed in Greece and Albania, sublineages L2 and L3 in nearby Bulgaria. Sublineage L4 is the most represented in available WGS data with twelve strains. In addition, the Eremenko et al. investigation contributed 21 additional sublineage L4 strains, from European Russia (20 strains) and Far East Siberia (one strain) (Eremenko et al., 2021). The Tsiankovskii lineage appears to be strongly associated with Eastern Europe and European Russia. The presence of the lineage in West Kazakhstan could result from a single introduction from neighboring Russia at the north of the Caspian Sea.

#### The “Heroin” Lineage Is Present in Turkistan, South Kazakhstan, Along the Former Silk Road

**FIGURE 4:**
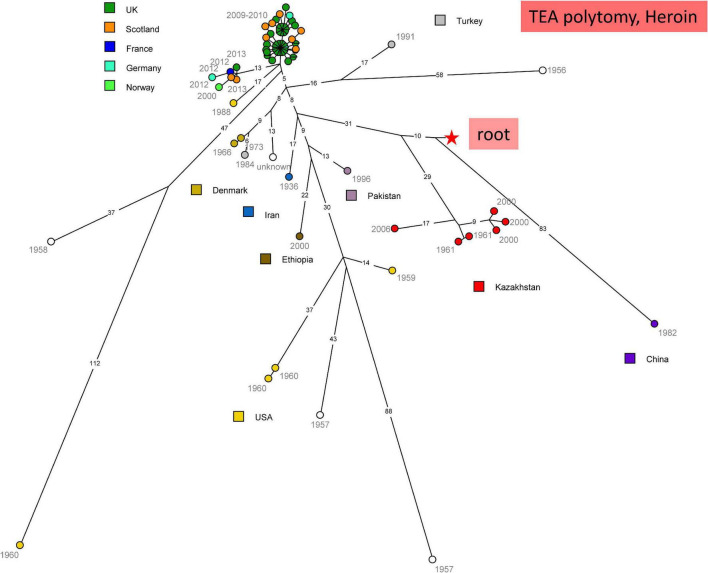
wgSNP analysis of strains assigned to TEA clade “Heroin.” Maximum parsimony analysis. Square root scaling. Branch lengths of five SNPs and more are indicated. 1018 SNPs in tree, tree size 1019 homoplasia 0.1%. Each strain is labeled with year of isolation. Color code indicates the country of isolation of the strains. The red star indicates the position of the root (MRCA) of the TEA polytomy.

Figure 4 shows the topology of the “Heroin” lineage evolution based upon all currently available WGS data. Starting from the root of the TEA polytomy marked by the red star, the deepest branching lineage is defined by a unique strain isolated in China. The strains isolated in Kazakhstan constitute a monophyletic lineage splitting ten SNPs later. The rest of the tree is composed predominantly of strains isolated in Europe or the United States. The heroin associated strains fall into two clusters (Hanczaruk et al., 2014). The true geographic origin of these strains is currently unknown, but the illicit opium or derived heroin itself was presumably produced in Afghanistan. With the exception of a single Ethiopian strain, the other non-European strains originate from Turkey, Pakistan and Iran, supporting the hypothesis that this part of the “Heroin” lineage is associated with Middle-East countries. All the “Heroin” strains from Kazakhstan were isolated in Turkistan, South Kazakhstan, along ancient terrestrial trade routes across central Asia (former “Silk Roads”).

#### Contributions to the Sterne/Ames, Vollum and B Clades

Seven strains (six from Kazakhstan and one from Kyrgyzstan) sequenced in the present project belong to the Sterne/Ames clade. Supplementary Figure 3 shows the relative position of the KAZ and KGZ strains with 31 additional strains of worldwide origins assigned to the A.Br.081 “Ames” group (Sahl et al., 2016). Available WGS data define a remarkable polytomy with six lineages radiating from a central point.

Lineage L1 contains the Ames lineage *stricto sensu* (Simonson et al., 2009). Strains KZ107 and KZ114 define a new closest branching point before the export to North America prior to early 19th century (Simonson et al., 2009). The topology of L1 clearly indicates that the unique published European strain from the Ames lineage, the Danish strain K670/88, is not an intermediate supporting an introduction of the Ames lineage in Texas *via* Europe as previously proposed (Derzelle et al., 2015). On the contrary, the two strains, the Danish strain and the Ames strain must result from two independent export events from East Asia, likely China. We propose that the Ames lineage was imported directly from China to Mexico at the time of the Manilla Galleons, part of the trade route between Asia and Spain in the 16th–18th century. The trade route connected Acapulco to Manilla (Philippines) and trans-Pacific merchandise (mostly from China) was distributed throughout the colony (Barragán-Álvarez, 2013). This trade was stopped with the decline in silver production in South and Central America and the independence of Mexico in 1821. Texas was Mexican until its 1845 annexation to the United States and anthrax was reported in Texas in 1860. Anthrax is present in Mexico but is likely underreported (Uribe and Fuentes, 1984; World Health Organization [WHO], 2008) and no information is available regarding the *B. anthracis* genotypes present in Mexico.

Two strains from two distinct events belong to the Vollum clade (Supplementary Figure 4). The strains collected 19 years apart in Turkistan (South Kazakhstan) and Atyrau are separated by a single SNP. They belong to a rare and deep-branching Vollum lineage comprising four strains. The vast majority of the more than 200 Vollum strains with public WGS data were isolated in the United States, often from imported goods such as Cashmere wool. The investigation of Vollum strains contaminating wool imported from the Indian subcontinent is in favor of a strong geographic association of Vollum with Kashmir (Derzelle et al., 2016).

The two B-clade sequenced strains from Kazakhstan are representatives from 12 strains from event 19 showing an identical MLVA31 genotype. One SNP separates the two strains. All twelve strains were isolated in North-East Kazakhstan (Pavlodar region, Ertis district) in 2016. Their closest neighbors are strains from Sakha-Yakutia, from the Yamal peninsula, and from the Altai Territory or adjacent Altai Republic (Timofeev et al., 2018, 2019; Eremenko et al., 2021). Supplementary Figure 5 shows the topology of the B-clade reflected by publicly available WGS data.

## Conclusion

We have presented here an extensive characterization of *B. anthracis* circulating in Kazakhstan. The TEA polytomy is largely predominant, particularly the “STI” sublineage, and to a much lower extend “Heroin” and “Tsiankovskii.” The “STI” sublineage appears to be remarkably specific for Kazakhstan and most sublineages are described here for the first time. This specificity is a strong indication that the STI lineage started to diversify at a time when the area constituted a geopolitical and economical entity which might have included the XinJiang region in China, i.e., after the dissolution of the Mongol empire in the 14th century, and before the conquest of the XinJiang region by China in the middle of the 18th century. In contrast, the topology of the “Tsiankovskii” detected in Kazakhstan is indicative of spillovers from European Russia. The strong geographic structuring observed for the different lineages constituting TEA would result from anthropic factors, including borders and trade routes.

## Data Availability Statement

The datasets presented in this study can be found in online repositories. The names of the repository/repositories and accession number(s) can be found below: NCBI bioproject PRJNA639508, run accessions, SRR12560170, SRR12633802, SRR12633803, and SRR16079441-SRR16079516.

## Author Contributions

GV edited the first version of manuscript. GV and AS conceptualized and designed the experiments. AS, J-PV, and GV analyzed the data. AS, AA, MK and YR genotyping and wrote the first version of the manuscript. LL and UI bacteriological researches and wrote the first version of the manuscript. All authors have read and approved the final version of the manuscript.

## Conflict of Interest

The authors declare that the research was conducted in the absence of any commercial or financial relationships that could be construed as a potential conflict of interest.

## Publisher’s Note

All claims expressed in this article are solely those of the authors and do not necessarily represent those of their affiliated organizations, or those of the publisher, the editors and the reviewers. Any product that may be evaluated in this article, or claim that may be made by its manufacturer, is not guaranteed or endorsed by the publisher.
