# Supplementary figures and images for: Bacillus anthracis Phylogeography: New Clues From Kazakhstan, Central Asia
Source: Front Microbiol. 2021 Dec 8;12:778225. doi: 10.3389/fmicb.2021.778225 (PMC8692834; doi:10.3389/fmicb.2021.778225)

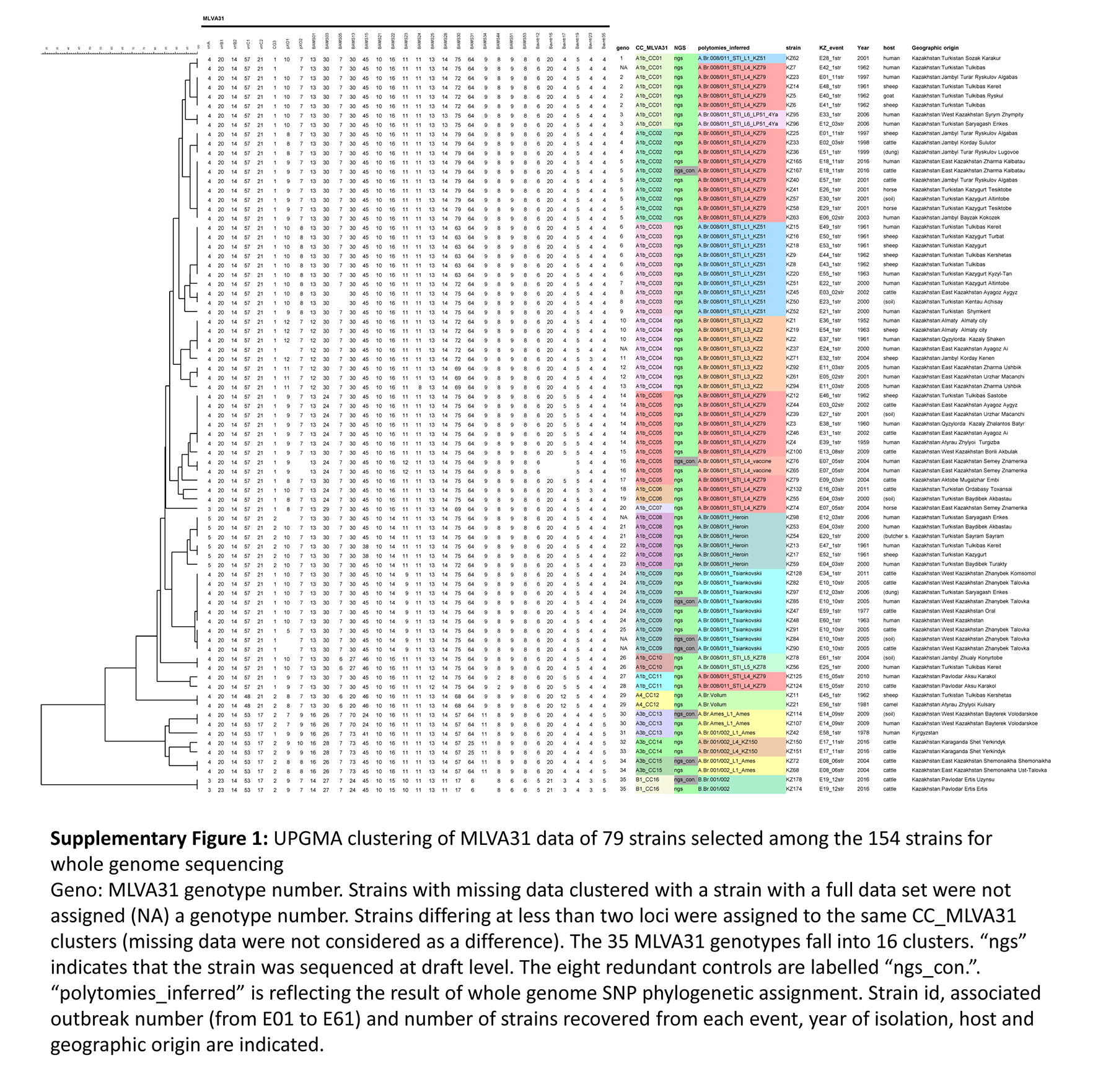

Supplement: Supplementary file 1 [file Image_1.tif]

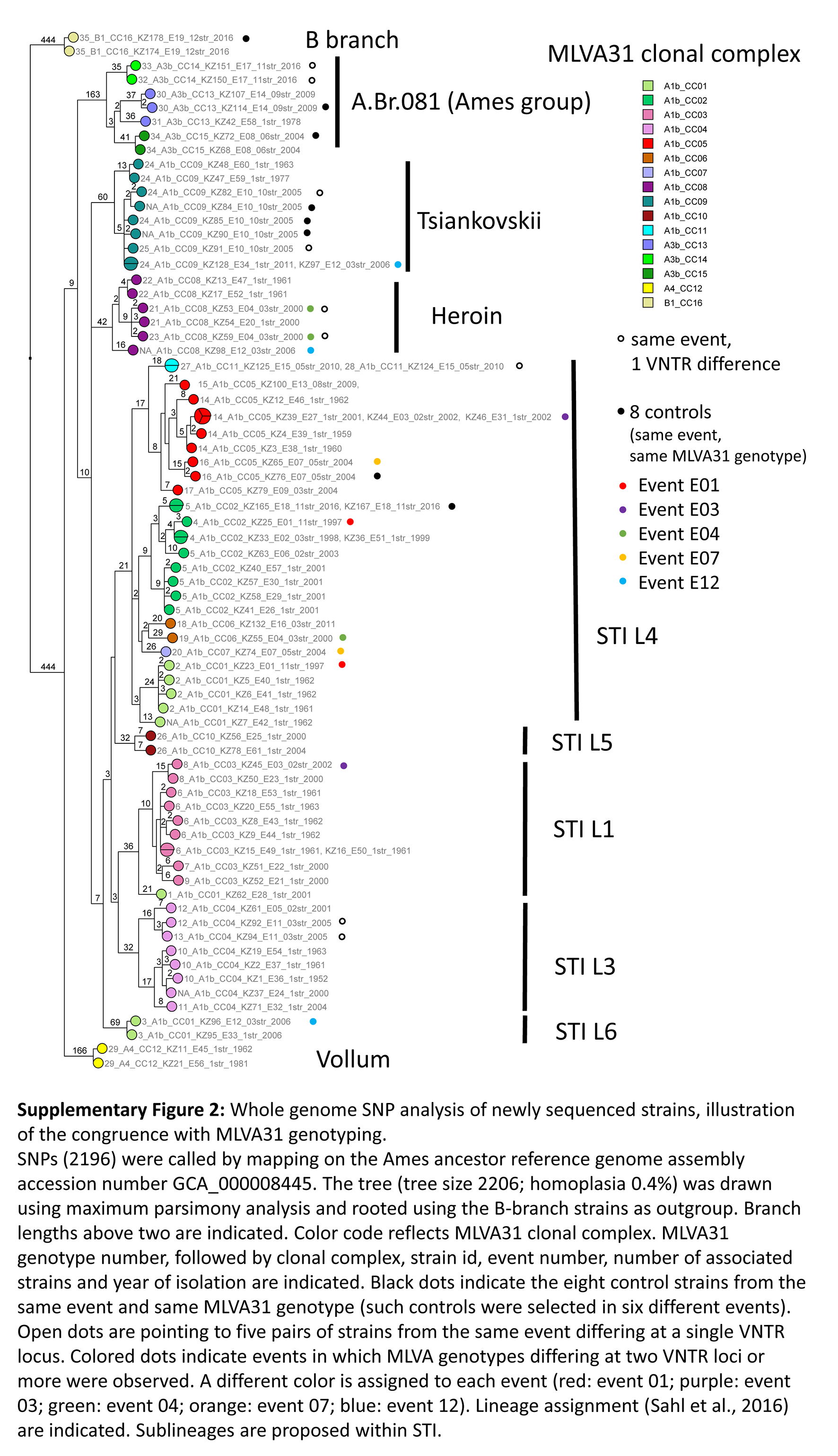

Supplement: Supplementary file 2 [file Image_2.tif]

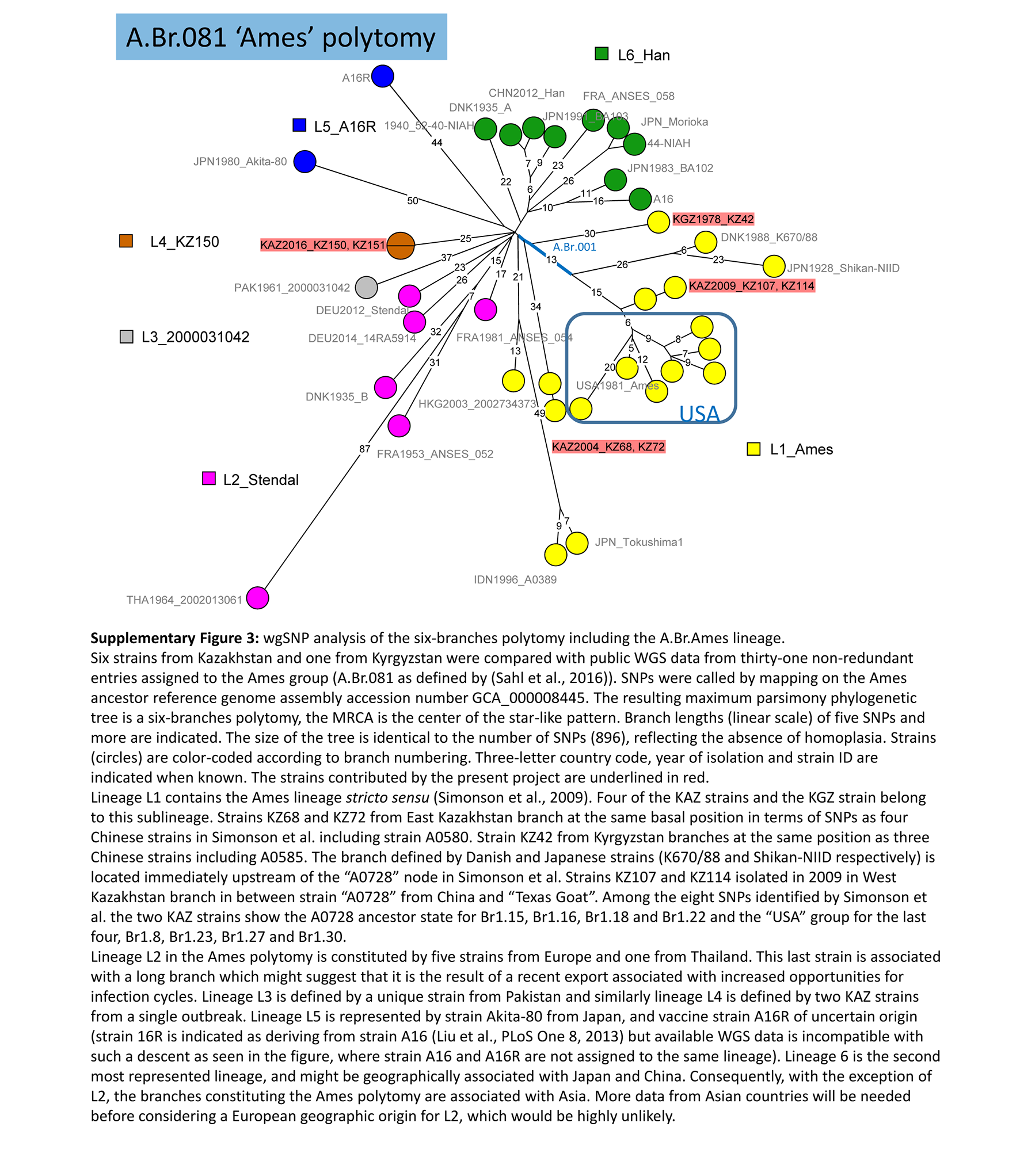

Supplement: Supplementary file 3 [file Image_3.tif]

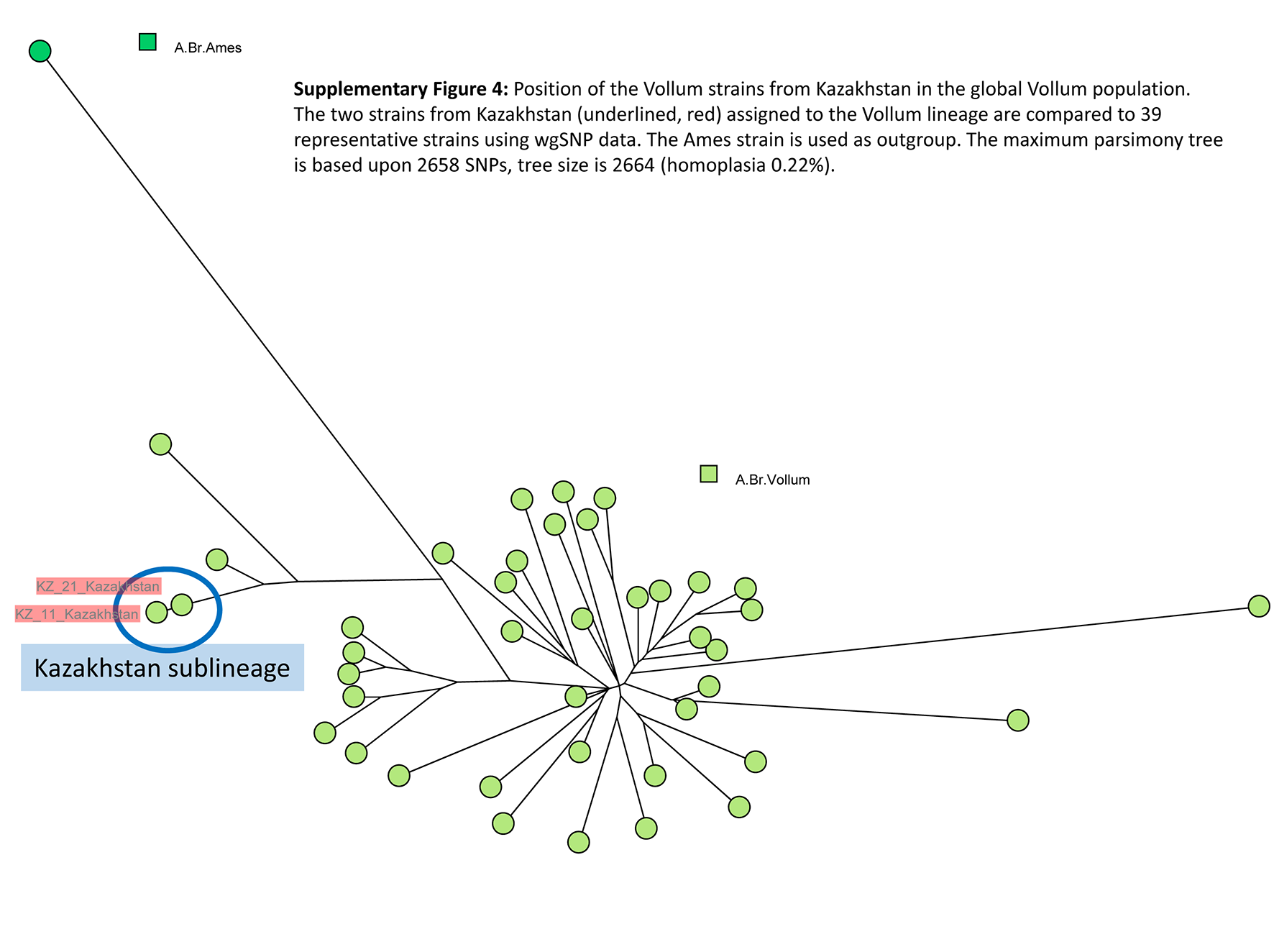

Supplement: Supplementary file 4 [file Image_4.tif]

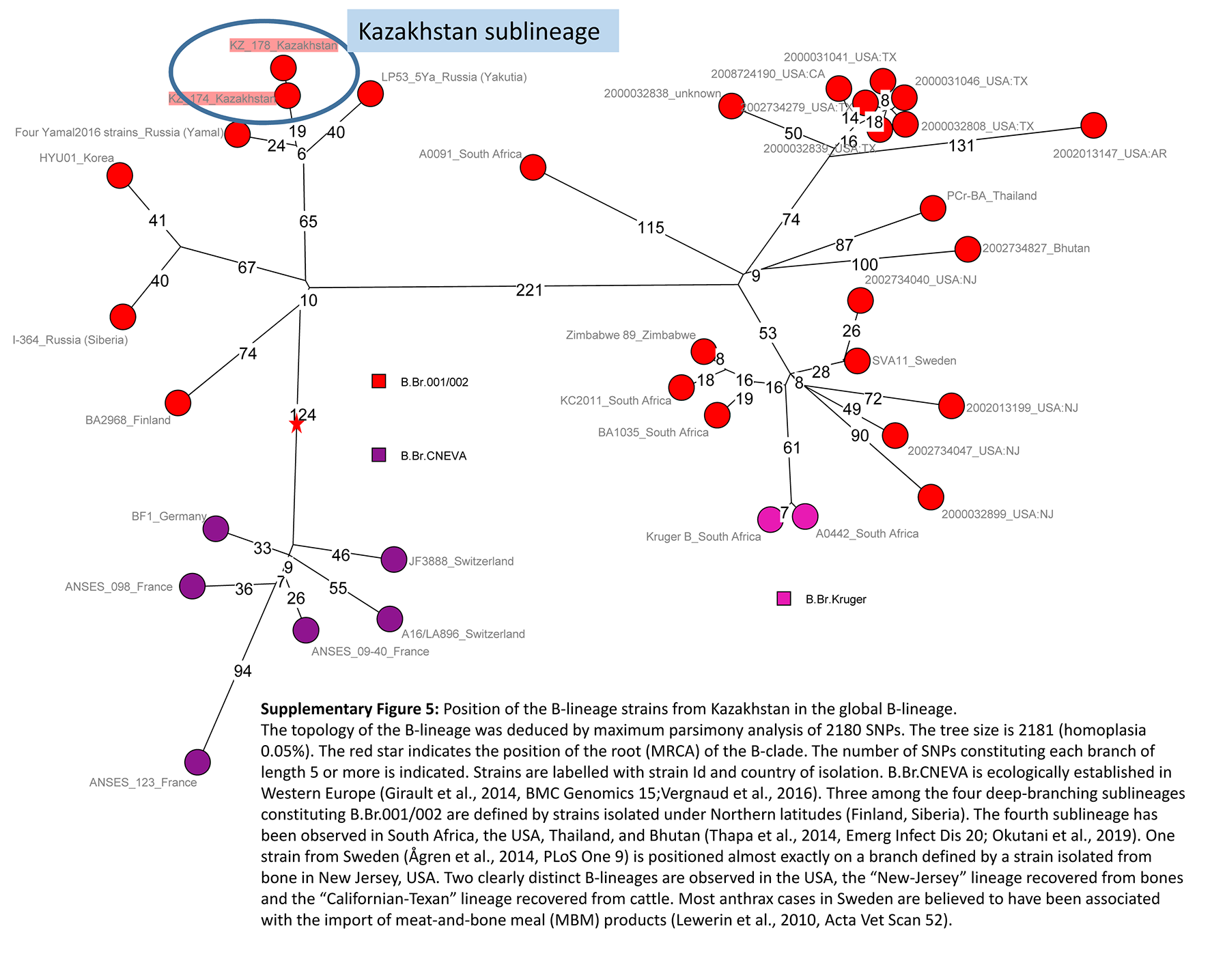

Supplement: Supplementary file 5 [file Image_5.tif]
