# Supplementary material for: Bacillus anthracis Phylogeography: New Clues From Kazakhstan, Central Asia
Source: Front Microbiol. 2021 Dec 8;12:778225. doi: 10.3389/fmicb.2021.778225 (PMC8692834; doi:10.3389/fmicb.2021.778225)
Supplement: Supplementary file 8 [file Table_3.pdf]

Available contextual information on 35 *Bacillus anthracis* outbreaks involving human cases, Kazakhstan, 1997-2016, and observed genetic diversity

Supplementary file for Shevtsov et al., *Bacillus anthracis* Phylogeography: New Clues From Kazakhstan, Central Asia. Front. Microbiol. 2021 12:778225.
doi: 10.3389/fmicb.2021.778225

E01_11str July 1997, Jambyl region, Turar Ryskulova district, Algabas village

On July 8th 1997 animals in an unvaccinated small livestock died of anthrax in Jambyl region, Turar Ryskulova district, Algabas village. On July 10-11, the livestock was vaccinated. During vaccination itself, 25 sheep died. The death of animals lasted until July 19, 1997. From a flock consisting of 775 heads of small cattle, six heads of cattle, one horse and a pig, 107 sheep and both the horse and pig died. On July 18, the material from several dead animals was sent to the laboratory. As a result of contact with animals, seven people developed cutaneous anthrax (with localization of carbuncles on the hands, forearms, lower leg) and subsequently recovered.

On July 18-19, two heads of small livestock and one horse from a nearby farm (500m away) were culled without veterinary examination. Part of the animal meat was sold in sausage manufactory of the Taraz city. Eight people involved in the butchering of animals developed cutaneous anthrax and were successfully treated.

The eleven strains which could be investigated fall in two MLVA31 genotypes 2 and 4 differing at two highly variable loci, pXO1 and BAMS30 (S1 Figure; strains KZ22 to KZ32 S1 Table; represented in Figure 1 by two strains, red dots). The available metadata does not allow inferring if the two grazing sites are each infected by one of the two lineages, of if both lineages are present in the same spot.

Event01 MLVA31 genotype 2, represented by KZ23, is less than five SNPs away from closest neighbors strains KZ5, KZ6 and KZ14 (from Events 40, 41 and 48 respectively) isolated in the Tulkibas district, Turkistan, in 1961-1962. The four strains share the same MLVA31 genotype 2 (S1 Figure and Figure 1). They constitute a sublineage of STI_Sublineage L4 (Figure 2).

Event01 MLVA31 genotype 4, represented by KZ25, is three SNPs away from closest neighbors KZ33 (Event02) and KZ36 (Event51) sharing the same MLVA31 genotype 4 isolated in 1998 and 1999 in Jambyl, Turar Ryskulov and Korday districts respectively. The three locations are well connected via the A-2 highway, going from Almaty to Tashkent along the Kyrgyzstan border. They constitute a sublineage of STI_Sublineage L4 (Figure 2).

E02_03str

In 1998 as a result of the culling of a sick cow in Jambyl, Korday, Sulutor, two people developed cutaneous anthrax. The treatment ended in recovery.

E03_02str February 2002, two different STI sublineages

On February 25, 2002, a worker involved in buying animal skins sought medical help in East Kazakhstan Ayagoz district. The diagnosis of anthrax has been clinically and laboratory confirmed. In the batch of skins, two skins were found that gave an Ascoli positive reaction, and a *B. anthracis* culture was isolated from each one.

Strains KZ44 (MLVA genotype 14) and KZ45 (MLVA genotype 8) from Event 03 differ at four VNTR loci (purple dots in Figure 1). The strains are separated by 120 SNPs. Each one of the strains was less than one SNP from strains recovered from independent events. KZ44 is identical in terms of wgSNP and MLVA31 genotypes to KZ39 (Event27) and KZ46 (Event31) and recovered from the same or an adjacent district a few months earlier or at the same time respectively. It is up to 16 SNPs away from three strains sharing MLVA genotype 14 and isolated in different regions in years 1959-1962 (Figure 2). The strains belong to STI Sublineage L4.

KZ45 is one SNP away from strain KZ50 (Event23) with identical MLVA31 genotype 8, isolated in Turkistan in year 2000. The strains belong to STI Sublineage L1 (Figure 2).

E04_03str July-August 2000, Turkistan, Baidibek, three strains from 'Heroin' (two) and 'STI' (one) sublineages.

In the Baidibek district between July 26th, 2000 and August 6th, 2000, in two anthrax reservoirs (the villages of Turakty and Akbastau) ten people who participated in the culling of farm animals (six cattle, one horse, four sheep) developed cutaneous anthrax. Carbuncles were localised on the hands (eight cases), on the lower leg (one case) and on the lower jaw (one case). The treatment ended in recovery.

Event04 (green dots, Figure 1) is similar to Event01 as it corresponds to the culling of animals from two nearby villages in the Baydibek district, Turkistan in year 2000. Three MLVA31 genotypes are observed, two of the corresponding strains (KZ53, KZ59) are very closely related (four SNPs difference, clonal complex CC08, genotypes 21 and 23, one VNTR difference, lineage "Heroin" S1 Figure) whereas the third (KZ55, clonal complex CC06, genotype 19, lineage STI Sublineage L4) is quite distinct (five or six VNTR loci difference, 100 SNPs away).

E05_02str

On June 2, 2001, two villagers participated in the culling of sick cattle. June 08, one of them turned to the clinic with a fever. He was diagnosed with an acute respiratory infection, and given antipyretics. On June 14, he again went to the hospital with complaints of swelling of the arm, and a carbuncle 1.5 x 2 cm in size on his right forearm. On June 7th the second resident observed suppuration in place of a skin cut between fingers 1 and 2. On June 15, he went to the hospital with a worsening condition and an increase in ulcers of up to 2 cm. During bacteriological examination, a culture of *B. anthracis* was isolated from the wounds in both patients. The treatment ended in recovery.

E06_02str

In September 2003, a sick cow was slaughtered and meat was sold to a local sausage manufacture. A few days later, three people participating in the slaughter of the animal

sought medical help with signs of a skin form of anthrax. The diagnosis was confirmed, the treatment ended in recovery. In the outbreak, appropriate epidemiological measures were taken with the elimination of products and disinfection.

E07_05str 2004, East-Kazakhstan, Semipalatinsk, Znamenka. STI vaccine strain recovered from human patients and not from sick horse

In June 14, 2004, a sick horse was culled. On June 15 horse meat was delivered to the sausage manufacture in the Semipalatinsk city. During the slaughter, one of the people had felt an insect bite in his hand. On June 18, a pustule appeared at the site of the bite. June 21, body temperature rose to 39 ° C, the pustule increased in size, headache, vomiting appeared, the back surface of the hand and the left side of the chest became very swollen, shortness of breath appeared. On June 24, he was hospitalized in a serious condition in the city infectious diseases hospital where he was diagnosed with anthrax, skin form, bullous variety complicated by anthrax sepsis, DIC, secondary reactive hepatitis, myocarditis. In the outbreak, another person was identified participating in the butchering of the horse with signs of anthrax. Also in the sausage manufacture where the carcass of the horse was delivered, a person involved in cutting meat fell ill. On June 28, he showed signs of fever and an ulcer on his hand. In the clinic, the surgeon washed the ulcer and prescribed antibiotic treatment. On July 3, the condition worsened and the patient was placed in an infectious diseases hospital where he was diagnosed with anthrax. Treatment of all patients ended in recovery.

The two strains recovered from the horse and blood-contaminated soil showed the same MLVA31 genotype 20, whereas the three strains recovered from the three human patients with cutaneous anthrax showed another, well distinct MLVA31 genotype 16, missing the three pXO2 associated VNTRs (orange dots, Figure 1). Whole genome SNP analysis confirms that the horse strain is unique within STI Sublineage L4 whereas the human isolates correspond to the STI vaccine. The lack of correspondence between the horse strain and the human patients is unexplained.

E08_06str 2004, East-Kazakhstan, sick cow one month after vaccination. Same genotype in all strains, A.Br.001/002_L1_Ames lineage.

On May 26, 2004, two residents of the Ust-Talovka village carried out a forced slaughter of a sick cow (vaccinated in April 2004). On June 2, one resident began to show signs of the disease: redness and itching of the right arm, followed by swelling of the arm and fever (38 °C). He entered the infectious disease department of the hospital on June 7. The patient expressed general toxic symptoms (fatigue, weakness, headache and malaise), fever and impaired activity of the cardiovascular system. He was diagnosed with anthrax, skin form, mixed bullous-erysipelas, severe course. The second resident had signs of anthrax on June 6: pain in the forearm and shoulder, fever up to 39 °C, swelling in the right elbow and a large bladder filled with serous-hemorrhagic fluid. On June 7, he was hospitalized in the infectious diseases ward. Based on the clinical and epidemiological data, the patient was diagnosed with anthrax, skin form, bullous-erysipelas, moderate severity. The disease ended in the recovery of patients.

E09_03str

On June 19, 2004, a sick unvaccinated cow was slaughtered. There were clinical signs of anthrax: epistaxis, enlarged spleen, tumor formation in the shoulder blade region. In seven people who participated in the slaughter the skin form of anthrax with localization of carbuncles on the hands was subsequently registered. The disease ended in the recovery of patients.

E10_10str

On August 15, five villagers made a forced slaughter of a sick bull. A few days later, they all fell ill with cutaneous anthrax. The diagnosis of Anthrax was made on the basis of clinical and epidemiological data, bacteriological and serological studies (test with anthraxin). Diseases of people ended in recovery. At this outbreak on August 14-24, nine cattle fell from anthrax.

E11_03str

On July 7, 2005, two residents of the village of Ush-Biik forcedly slaughtered a bull. The whole carcass was delivered to Ayagoz city. After five days, both villagers showed clinical symptoms of the cutaneous form of anthrax. A third patient was identified in Ayagoz city, where meat was transferred. The treatment ended in recovery.

E12_03str three different strains from three TEA lineages, “Tsiankovskii”, “Heroin” and “STI”

Event 12 occurred from September 11 to September 29, 2006 in the Enkes village, Saryagash district, Turkistan in 2006. Five reservoirs of anthrax were registered, in which nine people and five cattle fell ill. Four reservoirs were associated with forced slaughter of sick animals (cattle). The fifth was associated with the cutting of meat purchased in the market of Saryagash. Treatment of patients ended in recovery.Three strains were recovered. They showed a clearly different MLVA31 genotype, differing from each other at three VNTR loci. wgSNP analysis confirmed that three of the seven branches A.Br.008/011 (TEA008) polytomy (Sahl et al., 2016;Timofeev et al., 2019) are represented in this event, “Tsiankovskii”, “Heroin”, and “STI” (blue dots, Figure 1). This diversity is compatible with the epidemiological record, in which the independent slaughtering of sick animals and the purchase of meat in the nearby Saryagash market were mentioned.

E13_08str

In June 2009, four residents participated in the slaughter of a sick cow. A few days later they were hospitalized in an infectious diseases hospital with cutaneous anthrax. The treatment ended in recovery.

E14_09str

In June 2009, three residents participated in the slaughter of a sick cow. On June 18, they were hospitalized in an infectious diseases hospital with cutaneous anthrax. The treatment ended in recovery.

E15_05str

In June 2010, one active stationary-troubled community on anthrax was registered. Seven cases of people with anthrax were registered, of which two were fatal. Eight animals fell ill and died (cattle - 6, small livestock - 1, horse - 1).

E16_03str
On June 13, 2011, was carried out forced slaughter of a sick cow which in April was vaccinated against anthrax. Three people participated in the slaughter, and the meat was divided between 24 families of the village. After June 20, seven villagers were admitted to the infectious diseases department, where a skin form of anthrax was established. One hundred and twenty-one people who used meat were given prophylactic treatment. The remains of meat and carcasses were destroyed, and appropriate measures were taken in the outbreak. Treatment of patients ended in recovery.

E17_11str
In June 2016, in Shet district, in the village of Yerkindik, eight cases of human disease were recorded (two cases were fatal). Infection of people occurred during the forced slaughter of sick cows without veterinary inspection. The outbreak appeared in association with the erosion by heavy rains of the soil of an old reservoir of anthrax. The animals became infected by grazing.

E18_11str
Two people were contaminated during the forced slaughter in June 2016 of a cow without veterinary inspection. Treatment of people ended in recovery.

E19_12str
In 2016, in the village of Uzynsu, three cases of people with anthrax were registered. Of these, one case of the disease was fatal. Infection of people occurred during the forced slaughter of a cow, without veterinary examination. This is the first report of anthrax in animal or people in the village of Uzynsu.

E20
In year 2000, surface control in a butcher shop as part of epidemiological survey.

E21
On September 4th, 2000 in the meat pavilion of the Shymkent city market, a redness looking like a mosquito bite appeared on the cheek of a cleaning woman. On September 6th, small ulcers appeared on both cheeks, on September 10 appeared a fever, on September 12 the submandibular and cervical lymph nodes increased. The woman sought medical help on September 16 amid a worsening clinical condition (nausea, vomiting, temperature 40C, carbuncles 2x2 cm on both cheeks). She died on September 17th. The source of infection has not been established, because meat came to the market from all of Turkestan region.

E22
On August 31, 2000, a man carried out a forced slaughter of a sick calf. The liver and lungs were fried and eaten with a daughter. On September 2, clinical signs appeared in the form of multiple ulcers. On September 5, he was taken to the hospital in a coma, vomiting with an admixture of blood was noted. Three hours later, the patient died. The diagnosis of anthrax was laboratory confirmed.

E23

On June 16-17, 2000, three people who participated in the slaughter of a sick cow sought medical help. The disease ended in recovery. Confirmed cutaneous anthrax. *B. anthracis* culture was isolated from soil samples from the slaughter place.

E24

On November 31, 2000, during the forced slaughter of a sheep from a private household, one person was infected. Disease was fatal because of late application for medical help.

E25

From 07/03/2000 to 07/25/2000, 12 people became ill with anthrax as a result of their participation in the slaughter and treatment of two forcibly slaughtered cattle with anthrax. Everyone was diagnosed with a skin form, the disease ended in recovery.

E26

In July 2001, after a forced slaughter of a horse, five people who participated in butchering developped cutaneous anthrax. The disease ended in recovery.

E27

In June 2001, two cases of people with cutaneous anthrax were registered. Infection occurred during the forced slaughter of a cow from a private household. Disease of people ended in recovery.

E28

On August 23, 2001, a sick cow was slaughtered. On September 6, one of the participants in the slaughter sought medical help with clinical signs of a cutaneous form of anthrax. The treatment ended in recovery.

E29

In July 2001, after the forced slaughter of sick animals (one cow, one horse), six people fell ill with a skin form of anthrax, the disease ended in recovery.

E30

In July 2001, two men participated in the forced slaughter of a sick horse from their own farm. Horse meat, head and skin were delivered to the central market of Shymkent. During veterinary control, meat samples in the Ascoli reaction gave a positive result for anthrax. In one of the men, a wound and sores were found on the thumb of his right hand. The patient was hospitalized in the Shymkent city infectious diseases hospital with a diagnosis of anthrax. The diagnosis was confirmed by a skin allergy test. The disease ended in recovery.

E31

On February 16, 2002, a sick cow was forced to be slaughtered; meat was sold in a local cafe. On February 26, one of the participants in the slaughter sought medical help, the diagnosis of anthrax was clinically and laboratory confirmed, the disease ended in recovery. Epidemiological and epizootological measures were carried out in the outbreak; *B. anthracis* strain was isolated from the animal's skin.

E32

In August 2004, the deaths of more than 30 small livestock were recorded. In the summer of 2004, a flock of sheep grazed 27 km from the village in the place of Oi-Zhailau, where in 1987-1991, 2003, 2004, archaeological excavations of burials of the Bronze Age were carried out. The excavation area for all years was no more than 2000 m2. In the summer of 2004, a layer of land 20 cm deep was removed in an area of 400 m2. According to the owner of the flock, until 2004 farm animals did not graze in this tract. Sheep disease began on August 14 and within two weeks 10 sheep fell. Then the flock was taken about 50 km from the place. The death of animals continued on September 8 when another 17 heads of sheep fell. The disease arose suddenly, the death of animals occurred within 2-3 hours after the first signs of the disease. A culture of *B. anthracis* was isolated from the internal organs of a fallen lamb. The territory of the Kenen rural district has long been troubled for anthrax, more than 10 epizootic reservoir have been recorded here, the area of which is 4 km2, and cases of the disease have been recorded since 1962.

E33

In June 2007, two villagers conducted a forced slaughter of a sick cow. July 13, they entered the infectious diseases hospital. The first with a diagnosis “ anthrax, localized form, bullous form of the forearm and shoulder of the right hand, carbuncle 2 fingers of the left hand, severe form”. The second with a diagnosis “anthrax, localized skin form, carbuncle of the middle third of the left forearm, mild course”. The treatment ended in recovery.

E34

On July 20, 2011 a shepherd culled a cow. The meat was used to prepare food. As a result of the slaughter of the animal and cutting of meat three people fell ill with a skin form of anthrax. The treatment ended in recovery.

E35

In May 2016, a sick cow was slaughtered in the village. Three people involved in the slaughter fell ill with anthrax. Disease of people ended in recovery.
