# Supplementary material for: Bacillus anthracis Phylogeography: New Clues From Kazakhstan, Central Asia
Source: Front Microbiol. 2021 Dec 8;12:778225. doi: 10.3389/fmicb.2021.778225 (PMC8692834; doi:10.3389/fmicb.2021.778225)
Supplement: Supplementary file 9 [file Table_4.DOCX]

**Supplementary Figures Legends**

**Figure S1:** UPGMA clustering of MLVA31 data of 79 strains selected for whole genome sequencing

For clarity, when more than one strain from the same event shared an identical MLVA31 genotype, only one strain was kept (seventy-two strains were retained among the 154). Geno: MLVA31 genotype number. Strains with missing data clustered with a strain with a full data set were not assigned (NA) a genotype number. Strains differing at less than two loci were assigned to the same CC_MLVA31 clusters (missing data was not considered as a difference). The 35 MLVA31 genotypes fall into 16 clusters. “ngs” indicates that the strain was sequenced at draft level. “polytomies_inferred” is reflecting the result of whole genome SNP phylogenetic assignment. Strain id, associated outbreak number (from E01 to E61) and number of strains recovered from each event, year of isolation, host and geographic origin are indicated.

**Figure S2:** Whole genome SNP analysis of newly sequenced strains, illustration of the congruence with MLVA31 genotyping.

SNPs (2196) were called by mapping on the Ames ancestor reference genome assembly accession number GCA_000008445. The tree (tree size 2206; homoplasia 0.4%) was drawn using maximum parsimony analysis and rooted using the B-branch strains as outgroup. Branch lengths above two are indicated. Color code reflects MLVA31 clonal complex. MLVA31 genotype number, followed by clonal complex, strain id, event number, number of associated strains and year of isolation are indicated. Black dots indicate the eight control strains from the same event and same MLVA31 genotype (such controls were selected in six different events). Open dots are pointing to five pairs of strains from the same event differing at a single VNTR locus. Colored dots indicate events in which MLVA genotypes differing at two VNTR loci or more were observed. A different color is assigned to each event (red: event 01; purple: event 03; green: event 04; orange: event 07; blue: event 12). Lineage assignment (Sahl et al., 2016) are indicated. Sublineages are proposed within STI.

**Figure S3:** wgSNP analysis of the six-branches polytomy including the A.Br.Ames lineage.

Six strains from Kazakhstan and one from Kyrgyzstan were compared with public WGS data from thirty-one non-redundant entries assigned to the Ames group (A.Br.081 as defined by (Sahl et al., 2016)). One thousand and nine SNPs were called by mapping on the Ames ancestor reference genome assembly accession number GCA_000008445. The resulting maximum parsimony phylogenetic tree is a six-branches polytomy, the MRCA is the center of the star-like pattern. Branch lengths (linear scale) of five SNPs and more are indicated. The size of the tree is identical to the number of SNPs, reflecting the absence of homoplasia. Strains (circles) are color-coded according to branch numbering. Three-letter country code, year of isolation and strain ID are indicated when known. The strains contributed by the present project are underlined in red. Within the L1_Ames lineage, the A.Br.001 branch (Sahl et al., 2016) is shown in blue. Strains from Texas, USA, including the Ames strain, are boxed (Ames lineage stricto sensu).

Lineage L1 contains the Ames lineage *stricto sensu* (Simonson et al., 2009). Four of the KAZ strains and the KGZ strain belong to this sublineage. Strains KZ68 and KZ72 from East Kazakhstan branch at the same basal position in terms of SNPs as four Chinese strains in Simonson et al. including strain A0580. Strain KZ42 from Kyrgyzstan branches at the same position as three Chinese strains including A0585. The branch defined by Danish and Japanese strains (K670/88 and Shikan-NIID respectively) is located immediately upstream of the “A0728” node in Simonson et al. Strains KZ107 and KZ114 isolated in 2009 in West Kazakhstan branch in between strain “A0728” from China and “Texas Goat”. Among the eight SNPs identified by Simonson et al. the two KAZ strains show the A0728 ancestor state for Br1.15, Br1.16, Br1.18 and Br1.22 and the derived “USA” group boxed in Figure S3 for the last four, Br1.8, Br1.23, Br1.27 and Br1.30.

Lineage L2 in the Ames polytomy is constituted by five strains from Europe and one from Thailand. This last strain is associated with a long branch which might suggest that it is the result of a recent export associated with increased opportunities for infection cycles. Lineage L3 is defined by a unique strain from Pakistan and similarly lineage L4 is defined by two KAZ strains from a single outbreak. Lineage L5 is represented by strain Akita-80 from Japan, and vaccine strain A16R of uncertain origin (strain 16R is indicated as deriving from strain A16 (Liu et al., 2013) but available WGS data is incompatible with such a descent as seen in the figure, where strain A16 and A16R are not assigned to the same lineage). Lineage 6 is the second most represented lineage, and might be geographically associated with Japan and China. Consequently, with the exception of L2, the branches constituting the Ames polytomy are associated with Asia. More data from Asian countries will be needed before considering a European geographic origin for L2, which would be highly unlikely given the geographic association of all other lineages with Asia. The more likely explanation is that L2 is also of Asian origin.

**Figure S4:** Position of the Vollum strains from Kazakhstan in the global Vollum population.

The two strains from Kazakhstan assigned to the Vollum lineage are compared to 39 representative strains using wgSNP data. The Ames strain is used as outgroup. The maximum parsimony tree is based upon 2658 SNPs, tree size is 2664 (homoplasia 0.22%).

**Figure S5:** Position of the B-lineage strains from Kazakhstan in the global B-lineage.

The topology of the B-lineage was deduced by maximum parsimony analysis of 2180 SNPs. The tree size is 2181 (homoplasia 0.05%). The red star indicates the position of the root (MRCA) of the B-clade. The number of SNPs constituting each branch is indicated. Strains are labelled with strain Id and country of isolation. Current knowledge separates the B-clade in B.Br.CNEVA and B.Br.001/002. B.Br.CNEVA is ecologically established in Western Europe, and particularly the Alps (Girault et al., 2014;Vergnaud et al., 2016). B.Br.001/002 is geographically much more wide spread. Three among the four deep-branching sublineages constituting B.Br.001/002 are defined by strains isolated under Northern latitudes (Finland, Siberia). The fourth sublineage is strikingly different in terms of geographic spread and branch lengths. It has been observed so far in South Africa, but also in the USA, Thailand, and Bhutan (Thapa et al., 2014;Okutani et al., 2019). One strain from Sweden (Ågren et al., 2014) is positioned almost exactly on a branch defined by a strain isolated from bone in New Jersey, USA. Two clearly distinct B-lineages are observed in the USA, the “New-Jersey” lineage and the “Californian-Texan” lineage. Interestingly, whereas the second lineage was recovered in California from cattle, suggesting that it is ecologically established, the “New-Jersey” lineage was recovered from bones. Most anthrax cases in Sweden are believed to have been associated with the import of meat-and-bone meal (MBM) products (Lewerin et al., 2010), which would fit with the observed topology and suggests that the Swedish and New-Jersey strains share a recent common origin. Specific historical research on the source of Swedish as well as New Jersey MBM imports would be needed to source more precisely the contamination events. The presence of a very long branch suggests that the fourth B-sublineage encountered great opportunities in terms of infection cycles per year, reminiscent of the fast WNA lineage expansion observed in North America. The geographic places where this fourth sublineage is currently present, including South Africa, would not reflect the ancestral location of B.Br.001/002.

**References:**

Ågren, J., Finn, M., Bengtsson, B., and Segerman, B. (2014). Microevolution during an Anthrax outbreak leading to clonal heterogeneity and penicillin resistance. *PLoS One* 9**,** e89112.

Girault, G., Blouin, Y., Vergnaud, G., and Derzelle, S. (2014). High-throughput sequencing of *Bacillus anthracis* in France: investigating genome diversity and population structure using whole-genome SNP discovery. *BMC Genomics* 15**,** 288.

Lewerin, S.S., Elvander, M., Westermark, T., Hartzell, L.N., Norstrom, A.K., Ehrs, S., Knutsson, R., Englund, S., Andersson, A.C., Granberg, M., Backman, S., Wikstrom, P., and Sandstedt, K. (2010). Anthrax outbreak in a Swedish beef cattle herd--1st case in 27 years: Case report. *Acta Vet Scand* 52**,** 7.

Liu, X., Wang, D., Ren, J., Tong, C., Feng, E., Wang, X., Zhu, L., and Wang, H. (2013). Identification of the immunogenic spore and vegetative proteins of *Bacillus anthracis* vaccine strain A16R. *PLoS One* 8**,** e57959.

Okutani, A., Inoue, S., and Morikawa, S. (2019). Comparative genomics and phylogenetic analysis of *Bacillus anthracis* strains isolated from domestic animals in Japan. *Infect Genet Evol* 71**,** 128-139.

Sahl, J.W., Pearson, T., Okinaka, R., Schupp, J.M., Gillece, J.D., Heaton, H., Birdsell, D., Hepp, C., Fofanov, V., Noseda, R., Fasanella, A., Hoffmaster, A., Wagner, D.M., and Keim, P. (2016). A *Bacillus anthracis* Genome Sequence from the Sverdlovsk 1979 Autopsy Specimens. *MBio* 7.

Simonson, T.S., Okinaka, R.T., Wang, B., Easterday, W.R., Huynh, L., U'ren, J.M., Dukerich, M., Zanecki, S.R., Kenefic, L.J., Beaudry, J., Schupp, J.M., Pearson, T., Wagner, D.M., Hoffmaster, A., Ravel, J., and Keim, P. (2009). *Bacillus anthracis* in China and its relationship to worldwide lineages. *BMC Microbiol* 9**,** 71.

Thapa, N.K., Tenzin, Wangdi, K., Dorji, T., Migma, Dorjee, J., Marston, C.K., and Hoffmaster, A.R. (2014). Investigation and control of anthrax outbreak at the human-animal interface, Bhutan, 2010. *Emerg Infect Dis* 20**,** 1524-1526.

Vergnaud, G., Girault, G., Thierry, S., Pourcel, C., Madani, N., and Blouin, Y. (2016). Comparison of French and Worldwide *Bacillus anthracis* Strains Favors a Recent, Post-Columbian Origin of the Predominant North-American Clade. *PLoS One* 11**,** e0146216.
